# Epidemiology of rheumatic diseases in indigenous populations in Latin-Americans

**DOI:** 10.1007/s10067-016-3298-6

**Published:** 2016-05-14

**Authors:** Ingris Peláez-Ballestas, Bernardo A. Pons-Estel, Rubén Burgos-Vargas

**Affiliations:** Rheumatology Department, General Hospital of México, Dr. Eduardo Liceaga, Dr. Balmis 148. Colonia Doctores. Del. Cuauhtemoc, C.P. 06726 Mexico, Mexico; Hospital Provincial de Rosario, Leandro N. Alem 1450, Rosario, Santa Fe Argentina; Faculty of Medicine, Rheumatology Department, Hospital General de México, Universidad Nacional Autonoma de México, Mexico, Mexico

During the last few years, we have achieved a good deal of knowledge on the epidemiology of the rheumatic diseases in the world. Along with recent studies on the prevalence of rheumatic diseases such as osteoarthritis (OA), regional pain syndromes (RRPS), rheumatoid arthritis (RA), spondyloarthritides (SpA) mainly ankylosing spondylitis (AS) and psoriatic arthritis (PsA), fibromyalgia, gout, and some of the connective tissue diseases, we have learned about risk factors for the occurrence of these disorders in the community.

The prevalence of rheumatic diseases and related musculoskeletal disorders worldwide is very high, but when we look at the prevalence of each disorder, we find variations in their rank order [1, 2]. For example, OA and RPPS rank at the top in most studies, but their prevalence might vary from 2.3 % in México [3] to 20.4 % in Cuba [4]. Regarding RA, its prevalence goes from 0.17 % in the Philippines [5] to 1.49 % in México [3].

These phenomena may be explained in different ways. Perhaps, the most important relates to the study itself: sampling, case index definition, diagnostic and classification criteria, study design, and methodology. The use of homogeneous designs and definitions for epidemiological studies might solve such differences. In this sense, we have followed other studies in using the Community Oriented Program for the Control of Rheumatic Diseases (COPCORD) program for the detection of individuals with musculoskeletal (MSK) diseases. Then, we have followed universal classification criteria for each disease. In this way, we have minimized the role of study design in disease prevalence variations.

As a group, rheumatic and other MSK diseases cause a huge socioeconomic burden to patients and their household, society, and the country indeed. Yet, their relevance is often minimized, particularly in developing nations, countries with fragmented health care system, and poor nations [6]. Latin America, a geographic area that includes 26 countries, is plagued by these and more local problems that make the care of rheumatic patients certainly difficult.

The approach of the studies presented here and in previous papers has been dimensioning the magnitude of the problem by investigating the prevalence of various rheumatic diseases, their cost, effect on functioning, impact in quality of life as well as their clinical features. Our previous publications, in particular, a multilevel analysis of OA [7] and RA [8] have shown that speaking an indigenous language is associated with the prevalence of RA. It is therefore that this approach mainly focuses on some Latin American native populations.

It is undisputed that statistics and solid data that provide information about the incidence, prevalence, mortality, and impact of these conditions on the indigenous peoples of Latin America are needed. As a response to this regional challenge, a Latin American group called GLADERPO (Grupo Latino Americano de estudio De Enfermedades Reumaticas en Pueblos Originarios) was created. This group complies with the objective of carrying out epidemiological, genetic, and anthropological studies related to rheumatic diseases in indigenous peoples of Latin America. This project is placed within the framework of this regional effort.

Now, we present in this volume of *Epidemiology of rheumatic diseases in indigenous populations in Latin-Americans* the results of a large study on the prevalence of rheumatic disease in various indigenous populations in Latin America.

Noteworthy, we kept respect with the word and the concept behind of “indigenous” people to name the population which first originated in a particular land. The identification of these people has been a constant and serious problem worldwide. For example, the indigenous population of Australia is called “Aboriginal,” in Canada “aboriginal people,” in the USA “American Indians” and “Alaskan Natives.” In Argentina, Venezuela, and Mexico, the denomination is “indigenous” or “original” [9–11].

Nonetheless, the debate is far beyond people’s name; it also includes the denominations given to indigenous, governmental, non-governmental, and academic institutions as well as the use of language and self-recognition as variables that define indigenous people [10]. The identification of indigenous people is problematic; certain information, for example, that related on the effect of health care among indigenous groups in Mexico and other Latin American countries is scarce [11]. In this study, we have therefore relied on auto-identification criteria as well as framework of law pertaining to each participating country.

The populations included in this study was the Maya-Yucateco, Mixtec, Chontal, and Rarámuri from Mexico; Warao, Kari’ña, and Chaima from Venezuela; and Qom from Argentina. The design of the study, including sampling, case definition, and methodology, were similar to all studies. Basically, sampling was a “census,” case definition was “individuals with musculoskeletal pain, stiffness or swelling in the last 7 days and/or at any point during their lifetime,” and methodology was that proposed by COPCORD.

In this sense, the information presented here is unique since it includes a total of 6155 indigenous people from eight communities in three countries assessed by the same questionnaire and evaluated by current classification criteria and expert’s opinion.

Overall, low back pain (13.3 %; 95%CI 12.4–14.2), OA (9.7 %; 95%CI 8.9–10.4), and RPPS (5.9 %; 95%CI 5.3–6.6) were the most prevalent rheumatic disease across all populations. Regarding some examples showing wide variations in the prevalence of certain diseases among the different populations, the prevalence of RA was 2.4 % in Qom and 0.4 % in Mixtec, low back pain 19.8 % in Qom and 1.5 % in Rarámuri, and OA in 32.1 % in Chontal and 3.8 % in Qom. In Mixtec, the prevalence of MSK pain in the last 7 days or at any point during their lifetime occurred in 46.1 and 65.3 % but 20 and 14.4 % in Raramuris. Variations were not related to the design of the study but to the characteristics of the population and environmental factors, for example heavy loads in Chontales versus Rarámuris or Maya-Yucateco in people not exposed to the same environment.

Parallel anthropological studies have identified barriers preventing the access of indigenous population, particularly those with RA to medical care. The fact that the Mayan-Yucateco, Qom, and Warao are mostly monolingual hinders the communication with health providers. There are also geographical barriers that preclude many patients to benefit from health care in specialized centers and on the other hand the lack of institutional health universal coverage such as the Mexican populations [12].

The information gathered here may open a new set of possibilities to develop new and more advanced epidemiological and anthropological studies and particularly the design of interventional studies in the community for an early detection of the rheumatic diseases and generation of strategies to prevent their short and long-term consequences to implement public policies on musculoskeletal health and disability.
